# Recombinant vacuolar iron transporter family homologue PfVIT from human malaria-causing *Plasmodium falciparum* is a Fe^2+^/H^+^exchanger

**DOI:** 10.1038/srep42850

**Published:** 2017-02-15

**Authors:** Paola Labarbuta, Katie Duckett, Catherine H. Botting, Osama Chahrour, John Malone, John P. Dalton, Christopher J. Law

**Affiliations:** 1 School of Biological Sciences, Medical Biology Centre, Queen’s University Belfast, Belfast BT9 7BL, United Kingdom; 2 School of Biology, Biomedical Sciences Research Complex, University of St Andrews, North Haugh, St Andrews, Fife KY16 9ST, United Kingdom; 3 Spectroscopy Group, Analytical Services, Almac, 20 Seagoe Industrial Estate, Craigavon BT63 5QD, United Kingdom

## Abstract

Vacuolar iron transporters (VITs) are a poorly understood family of integral membrane proteins that can function in iron homeostasis via sequestration of labile Fe^2+^ into vacuolar compartments. Here we report on the heterologous overexpression and purification of PfVIT, a vacuolar iron transporter homologue from the human malaria-causing parasite *Plasmodium falciparum*. Use of synthetic, codon-optimised DNA enabled overexpression of functional PfVIT in the inner membrane of *Escherichia coli* which, in turn, conferred iron tolerance to the bacterial cells. Cells that expressed PfVIT had decreased levels of total cellular iron compared with cells that did not express the protein. Qualitative transport assays performed on inverted vesicles enriched with PfVIT revealed that the transporter catalysed Fe^2+/^H^+^ exchange driven by the proton electrochemical gradient. Furthermore, the PfVIT transport function in this system did not require the presence of any *Plasmodium*-specific factor such as post-translational phosphorylation. PfVIT purified as a monomer and, as measured by intrinsic protein fluorescence quenching, bound Fe^2+^ in detergent solution with low micromolar affinity. This study of PfVIT provides material for future detailed biochemical, biophysical and structural studies to advance understanding of the vacuolar iron transporter family of membrane proteins from important human pathogens.

Malaria is a serious parasitic disease, caused by unicellular protozoa of the genus *Plasmodium*, which is responsible for high morbidity and mortality rates among those infected. In 2013 alone the most virulent human malaria parasite, *P. falciparum*, was responsible for an estimated 198 million clinical cases of infection and more than 0.5 million deaths^1^. Emerging resistance against the therapies commonly used to control malaria infection means there is acute need to identify new therapeutic targets in the parasite^2^,^3^. In view of the importance of iron metabolism to survival of plasmodial cells, especially during the intraerythrocytic stage of the life cycle, membrane transporter proteins that function in iron homeostasis in the parasite could represent attractive novel drug targets^3^.

Iron is an essential micronutrient for almost all organisms and is a constituent of biological macromolecules involved in critical cellular processes such as energy production, respiration and DNA synthesis^4^,^5^. However, the same redox properties of iron that have been exploited for beneficial purposes also make it potentially cytotoxic; oxidation of excess ferrous iron (Fe^2+^) to the ferric state (Fe^3+^) by the Fenton reaction in the cell cytoplasm results in production of reactive free hydroxyl radicals that cause oxidative damage to nucleic acids, lipids and proteins^6^. To prevent such damage but at the same time ensure adequate supply of essential iron, cells have evolved integrated mechanisms for maintenance of iron homeostasis via tight and coordinated regulation of the systems that control iron provision and storage^4^. A major role in this regulatory process is played by integral membrane proteins that function in transport of ferrous iron^7^,^8^. One group of transporters involved in this process is the vacuolar iron transporter (VIT) family (Transporter Classification Database #2.A.89; www.tcdb.org), poorly understood proteins that function in secondary active transport of Fe^2+^(and also Mn^2+^ and, in some cases, Zn^2+^) across membranes, probably via a proton-driven antiport mechanism^9^. Although VIT family proteins have been studied mainly in plants^10^,^11^,^12^ and yeast^13^, homologues have also been identified in other eukaryotes (but not animals) and in bacteria and archaea^14^. VIT proteins are single polypeptides of between 250–400 amino acids organised into 5 putative transmembrane spanning α-helices (TMHs) and with an extended, hydrophilic N-terminal tail. In plant representatives of the VIT family the TMHs have a 2+2+1 arrangement, whereas bacterial and archaeal VIT homologues may have them in a 1+2+2 or 2+3 arrangement^9^ (Supplementary Fig. S1).

Bioinformatics studies performed by Martin *et al*.^15^,^16^ suggested a 273 amino acid, ~31 kDa VIT family homologue termed PfVIT (PlasmoDB ID: PF3D7_1223700; Supplementary Fig. S2a) was localised to the digestive vacuole (DV) membrane of *P. falciparum* from where it transports ferrous iron into the acidic vacuole interior via an antiport reaction. Although more recent experimental work by Slavic *et al*.^17^ implied that PfVIT is actually localised to the endoplasmic reticulum (ER) membrane of *Plasmodium*, it could not be established whether PfVIT functioned as an exchanger or facilitative transporter of Fe^2+^. To enable study of the transport mechanism of the protein, we exploited synthetic, codon-optimised DNA for the heterologous high-level expression of recombinant PfVIT in *Escherichia coli*, followed by purification of the recombinant transporter. Our results demonstrate that heterologous overexpression of PfVIT in *E. coli* confers increased resistance to iron-mediated cell death to the bacterial cells and that the PfVIT transport mechanism is Fe^2+^/H^+^ antiport driven by the proton electrochemical gradient.

## Results

### Heterologous expression of PfVIT using synthetic gene technology

**Figure 1:**
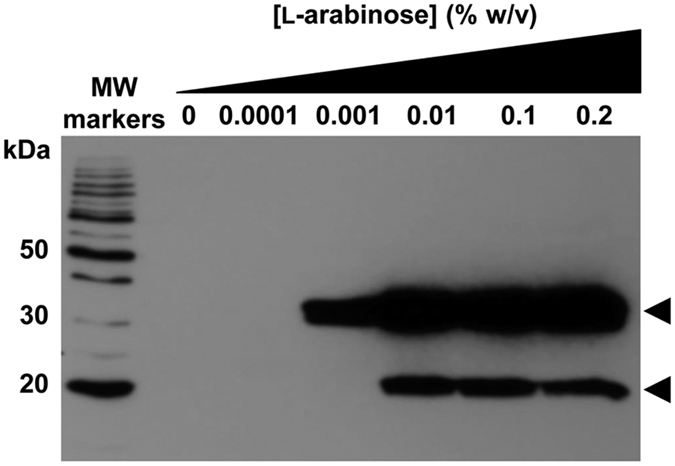
Western blot analysis of heterologous overexpression of recombinant *P. falciparum* PfVIT in *E. coli*. 80 μg of total protein from DDM-detergent solubilised membranes of *E. coli* LMG194 that overexpressed target protein from pBAD/*Myc*-His vector that contained synthetic, codon-optimised PfVIT coding sequence was loaded onto each lane of the gel. Hexahistidine-tagged PfVIT was detected by HisProbe-horseradish peroxidase. Cultures were grown at 25 °C for 4 h post addition of the indicated concentrations of L-arabinose inducer. Only an L-arabinose concentration of 0.001% w/v resulted in exclusive overproduction of full length recombinant PfVIT correctly targeted to the *E. coli* inner membrane. Upper arrow indicates ~32 kDa full length recombinant protein. Lower arrow indicates ~20 kDa truncated PfVIT.

Initial attempts to heterologously express full length *P. falciparum* VIT homologue, PfVIT, in several different expression strains of *E. coli* (including those that co-expressed tRNAs for *E. coli* rare codons) from pET or pBAD/*Myc*-His vectors that contained the native PfVIT coding sequence were unsuccessful. We therefore ligated a synthetic protein coding sequence that was optimised for heterologous expression in *E. coli* (Supplementary Fig. S3) into a modified pBAD/*Myc*-His A vector, transformed the resultant plasmid into *E. coli* LMG194, and tested the efficacy of this system for production of the target protein. A range of expression conditions were tested, including the effects of altering parameters such as growth temperature, L-arabinose inducer concentration, and time and length of induction on production of target protein. Western blot detection of the hexahistidine affinity tag of the protein construct in dodecyl-β-D-maltopyranoside (DDM)-detergent solubilised membrane fractions was used to determine conditions for successful overproduction. Although many of the conditions tested revealed heterologous expression of PfVIT, another band was also visible on western blots. Only one set of conditions resulted in exclusive production of full length PfVIT. As shown in Fig. 1, growth of test expression cultures at 25 °C for 4 h after addition of L-arabinose to a concentration of 0.001% w/v revealed a single band with an apparent molecular mass of ~32 kDa on the western blot. Increasing the L-arabinose concentration to 0.01% w/v or greater resulted in the appearance of a ~20 kDa band in addition to the ~32 kDa band. Although the apparent mass of the ~32 kDa band is about 10% smaller than the theoretical 34.9 kDa mass of the full length protein construct, anomalous migration on SDS-PAGE is a phenomenon commonly observed with membrane proteins^18^. There was no detectable expression of target protein in the absence of L-arabinose.

Mass spectrometry analyses of the ~32 kDa and ~20 kDa bands excised from a Coomassie-stained SDS-PAGE gel confirmed that the ~32 kDa band corresponded to full length PfVIT construct, whereas the ~20 kDa band corresponded to a product that likely arose due to *in vivo* proteolytic attack at the hydrophilic loop region that connects predicted transmembrane spanning helices 2 and 3 of the PfVIT protein (Supplementary Fig. S2b). Having established heterologous expression conditions that overproduced PfVIT and targeted it to the *E. coli* inner membrane, conditions were scaled-up to enable purification of recombinant transporter in the quantities required for downstream biochemical analysis.

### Heterologous expression of PfVIT in *E. coli* results in a phenotype with increased resistance to iron-mediated cell death

**Figure 2:**
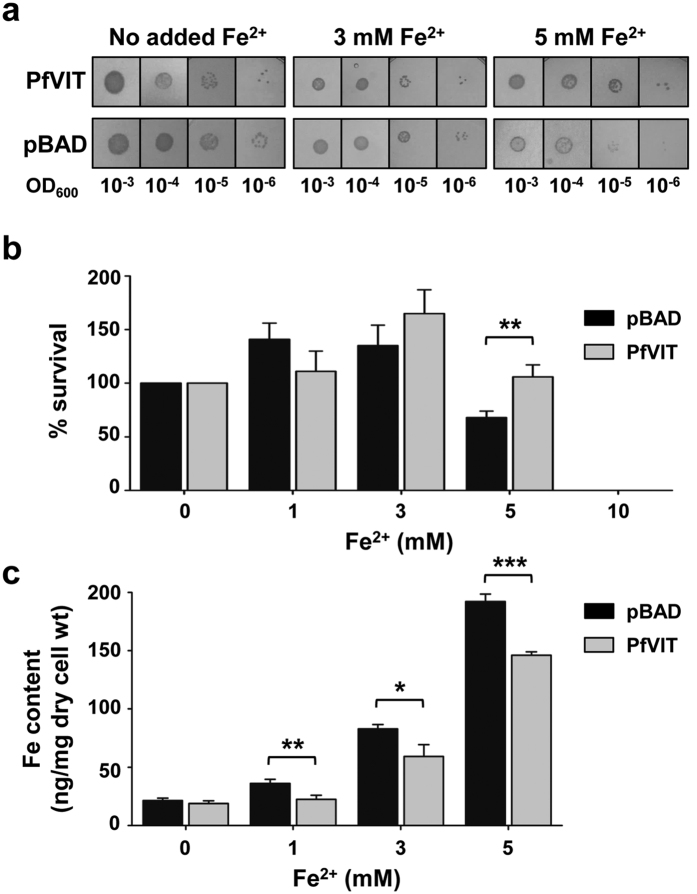
*E. coli* cells expressing PfVIT are more resistant to iron-mediated cell death and have reduced total iron content. (**a**) Growth of *E. coli* LMG194 cells transformed with pBAD vector encoding PfVIT on solid LB medium that contained Fe^2+^ (added in the form of ammonium FeSO_4_ in the presence of 2 mM ascorbic acid) at the concentrations indicated, and 0.001% w/v L-arabinose to induce expression of the protein. Cells transformed with empty pBAD vector were used as controls. Aliquots of 2 μl of a logarithmic dilution series of cells grown to an initial OD_600_ of 1.0 were spotted onto the solid medium and the plates incubated for 24 h at 30 °C. (**b**) The effect of functional expression of PfVIT on survival of *E. coli* LMG194 cells in liquid LB medium that contained Fe^2+^ at the concentrations indicated, and L-arabinose to induce expression of the protein. Cells transformed with empty pBAD vector provided controls. After 16 h incubation with shaking at 25 °C, cell viability was determined by performing colony forming unit (cfu) counts of cells plated onto solid LB medium. The cfu counts were normalised to those of cultures that were plated onto medium that contained no additional Fe^2+^. (**c**) *E. coli* LMG194 cells that overproduced recombinant PfVIT had reduced iron content compared to cells that harboured empty vector. Cells were exposed to the indicated concentrations of ferrous iron for 30 min prior to determination of total cellular iron content by ICP-MS. Data in (**b**) and (**c**) are the mean ± s.d. of three separate measurements, and significance was determined by unpaired, two-tailed Student’s *t*-test; *P < 0.05, **P < 0.01, ***P < 0.001.

Deletion of the gene encoding the vacuolar iron transporter CCC1 in the yeast *Saccharomyces cerevisiae* results in a mutant susceptible to iron toxicity due to an inability to transport excess intracellular iron into the vacuole^13^. The iron-sensitive phenotype of the *Δccc1* mutant can, however, be rescued by complementation with a plasmid that encodes PfVIT^17^ or homologous plant VIT proteins^10^,^12^,^19^. We hypothesised that transformation of *E. coli* cells with a plasmid that encoded PfVIT would confer the bacterial cells with greater resistance to iron-mediated cell death if the expressed protein was functional in the bacterial inner membrane. We initially performed qualitative assays in solid media that contained various concentrations of added Fe^2+^ to assess the effects of heterologous expression of PfVIT from pBAD/*Myc*-His A vector on *E. coli* growth phenotype (Fig. 2). On LB agar plates that contained either no added Fe^2+^ or Fe^2+^ added to 3 mM, control cells that harboured empty vector exhibited similar growth to those that expressed PfVIT (Fig. 2a). However, when the concentration of Fe^2+^ was increased to 5 mM, PfVIT-expressing cells grew better than the controls.

The ability of PfVIT to confer *E. coli* with greater tolerance to Fe^2+^ was quantitated using survival assays that employed colony-forming unit (cfu) counts. The quantitative data (Fig. 2b), which assessed the viability of cells cultured in Fe^2+^ -containing liquid LB media, corroborated the results of the qualitative growth assays. As expected for cells under stress of recombinant protein overproduction, the cfu count of PfVIT-expressing cells (10^9^ cfu/ml) grown in liquid medium that contained no additional Fe^2+^ was 10-fold lower than that of cells which harboured empty vector (10^10^ cfu/ml). To assess the viability of cells grown in media that contained added Fe^2+^, subsequent cfu counts were expressed as a percentage of these controls. As shown in Fig. 2b, at external Fe^2+^ concentrations of 1 mM and 3 mM, the viability of PfVIT-expressing cells and those that harboured empty vector did not differ significantly and the growth of both strains was actually enhanced compared to the controls. Enhancement of cell survival at these Fe^2+^ concentrations was likely due to the immediate availability to the cells of ferrous iron for use in respiratory chain proteins and as an electron donor, and both the bacterial strains were therefore able to take advantage of these non-limiting, yet non-lethal, concentrations of ferrous iron to promote growth. However, when the concentration of exogenous ferrous iron was increased to 5 mM, the effect of heterologous expression of PfVIT became readily apparent; in contrast to the results of the cell survival assays performed at the lower Fe^2+^ concentrations, at 5 mM Fe^2+^ the viability of cells that contained empty vector was significantly impaired (*P* = 0.0063) compared to that of PfVIT-expressing cells. Neither transformant was capable of survival in media that contained Fe^2+^ at 10 mM.

The quantitative assays were repeated to assess if heterologous expression of PfVIT could render *E. coli* resistant to the cytotoxic effects of other divalent transition metal cations (Mn^2+^, Zn^2+^, Ni^2+^ and Co^2+^) and also to provide some insight into the potential substrate specificity of PfVIT. The assays revealed that PfVIT did not confer tolerance to those transition metals at the concentrations tested (Supplementary Fig. S4).

To determine whether the enhanced survival of PfVIT-expressing cells grown in Fe^2+^−containing medium was due to depletion of intracellular iron by PfVIT transport activity, total iron content of cells was measured using inductively coupled plasma-mass spectrometry (ICP-MS)^20^. Following 30-min exposure to FeSO_4_, cells that expressed PfVIT had a significant reduction in total iron content compared to cells that did not express the protein; at 1 mM Fe^2+^ the reduction in cellular iron content was ~39% (*P* = 0.0092), and at Fe^2+^ concentrations of 3 mM and 5 mM in the medium, iron content was reduced by ~29% (*P* = 0.0197) and ~24% (*P* = 0.0003), respectively (Fig. 2c).

Taken together, the metal sensitivity and ICP-MS data are supportive of the notion that recombinant PfVIT overexpressed in *E. coli* is functional in the bacterial inner membrane and can confer the bacterium with increased tolerance to ferrous iron. Furthermore, the experiments demonstrated that *Plasmodium*-specific factors, such as phosphorylation by parasite kinases, were not required for this PfVIT function in the bacterial system.

### PfVIT mediates Fe^2+^/H^+^exchange in inverted vesicles

The survival assay and ICP-MS data implied that a PfVIT-mediated efflux activity transported excess cytoplasmic Fe^2+^ out of the bacterial cell. To test if PfVIT could mediate such transport via exchange of ferrous iron for protons, we measured the changes in luminal pH of PfVIT-enriched inverted membrane vesicles upon addition of the divalent metal cation substrate. This was achieved by monitoring the changes in fluorescence of a pH-sensitive probe, acridine orange, encapsulated within the vesicles. To inhibit oxidation and precipitation of the ferrous iron substrate, the vesicle mixture was maintained under constant nitrogen flush for the duration of the assay. Inverted vesicles prepared from cells that overproduced glycerol 3-phosphate/P_i_ antiporter GlpT (an *E. coli* membrane protein that does not bind or transport divalent metal cations^21^) from pBAD vector were used as a control. Expression levels of PfVIT and GlpT in their respective vesicles were similar, as assessed by western blot analysis (Supplementary Fig. S5a).

**Figure 3:**
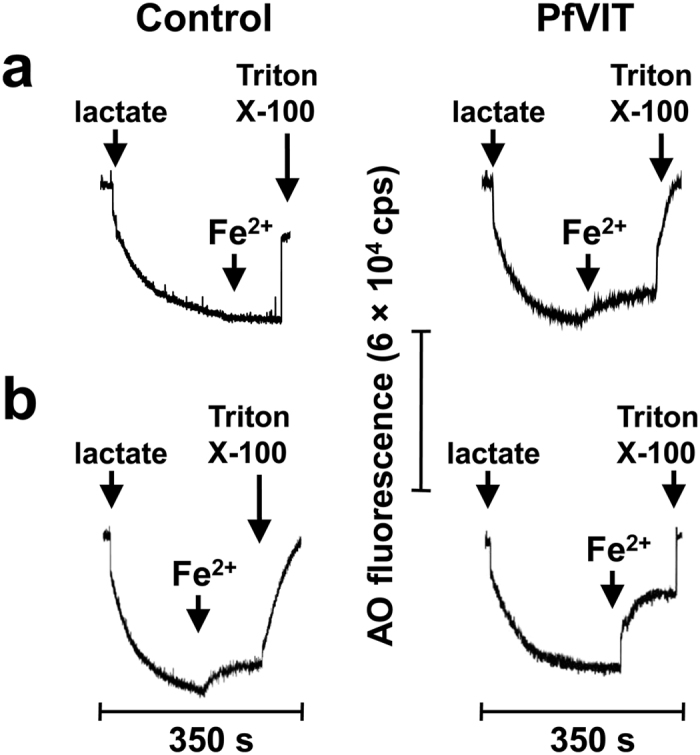
PfVIT-dependent Fe^2+^/H^+^ exchange in inverted vesicles. Measurements were performed by monitoring the fluorescence dequench of acridine orange upon addition of Fe^2+^ to inverted vesicles prepared from *E. coli* LMG194 cells that overproduced recombinant PfVIT (right traces) or, as a control, GlpT (left traces). Respiration-dependent generation of a transmembrane electrochemical gradient (acid inside) was established by addition of lactate as indicated. Once the quench of the fluorescent indicator reached a steady state, Fe^2+^ substrate was added to a final concentration of (**a**) 5 μM or (**b**) 50 μM. The pH gradient was dissipated by addition of Triton X-100 detergent at the time indicated. Traces are representative of experiments performed in triplicate on at least two separate preparations of vesicles. Fluorescence intensity was measured in counts per second (cps).

As shown in Fig. 3, addition of D-lactate to energise the vesicles resulted in quenching of the acridine orange fluorescence signal as protons were pumped by respiratory chain proteins into the vesicular lumen to generate a pH gradient (ΔpH; acid inside) across the vesicle membrane. Due to the oxygen-depleted conditions employed in the system to prevent oxidation and subsequent precipitation of the ferrous iron substrate, the scale of the respiration-induced acridine orange fluorescence quench in these assays was about an order of magnitude smaller than that observed in the assays that tested for transport of other metal cations, and which did not require anaerobic conditions (compare traces in Fig. 3 with those in Supplementary Fig. S5b–d). It should be noted that, in the oxygen-depleted system, carbonyl cyanide 3-chlorophenylhydrazone (CCCP) could not be used to dissipate the proton electrochemical gradient. This was due to a reaction that caused a precipitate to form and interfere with the spectroscopic measurements. Instead, Triton X-100 detergent was added to the mixture at the time indicated to disrupt the vesicle membranes and dissipate the ΔpH.

While vesicles prepared from cells that overexpressed PfVIT were clearly Fe^2+^ transport-active (as revealed by a slow dequench of the initial fluorescence signal upon addition of Fe^2+^ to 5 μM), control vesicles exhibited no transport activity (Fig. 3a). When the concentration of Fe^2+^ used to initiate transport was increased 10-fold to 50 μM, the negative control vesicles exhibited a small fluorescence dequench which could be attributed to activity of low-affinity, chromosomally encoded transporters capable of Fe^2+^ transport (Fig. 3b; left panel). In contrast, addition of 50 μM Fe^2+^ to PfVIT-enriched vesicles resulted in a much larger and instantaneous dequench of the acridine orange fluorescence (Fig. 3b; right panel). These results are in accord with a proton-driven Fe^2+^/H^+^ antiport function for PfVIT.

To test substrate specificity of PfVIT, the effects of addition of Mn^2+^, Ni^2+^ or Co^2+^ to the transport assay system were assessed (Supplementary Fig. S5b–d). Addition of Ni^2+^ or Co^2+^ to a final concentration of 5 mM (a concentration 1000-fold greater than the 5 μM concentration of Fe^2+^ that stimulated PfVIT-mediated transport) had no effect on the acridine orange fluorescence signal of PfVIT-containing vesicles. Addition of 5 mM Mn^2+^ to PfVIT-containing vesicles, however, did result in detectable transport activity (Supplementary Fig. S5b). Although this indicates that PfVIT possesses capacity to transport Mn^2+^ when the metal is present at relatively high and non-physiological concentrations, consideration of the results of the cell survival data presented in Supplementary Fig. S4a (in which there was no significant effect of PfVIT on cell survival at any of the Mn^2+^ concentrations tested) suggests that this transport is not biologically relevant and probably represents a low affinity interaction between PfVIT and Mn^2+^ in the inverted vesicle system. We therefore believe that our data are supportive of the contention by Slavic *et al*.^17^ that PfVIT is selective for Fe^2+^. We were unable to test if PfVIT possessed capacity to mediate proton-driven exchange of Zn^2+^, Cu^2+^, Mg^2+^ or Ca^2+^ in the same system due to interference from the activity of other transporters of these metals in the bacterial membrane^22^,^23^,^24^,^25^.

### Recombinant PfVIT purifies as a monomer

Recombinant PfVIT was purified to homogeneity using a two-column chromatography protocol. DDM-solubilised membranes were incubated with Co^2+^-affinity resin to bind the recombinant His-tagged protein. Elution of bound PfVIT by on-column thrombin cleavage typically yielded about 1.5 mg to 2 mg of semi-purified protein (from 12 l of culture) that migrated as a single fuzzy band with an apparent mass of ~30 kDa on Coomassie-stained SDS-PAGE (Supplementary Fig. S6). The protein was subsequently purified further by size-exclusion chromatography.

**Figure 4:**
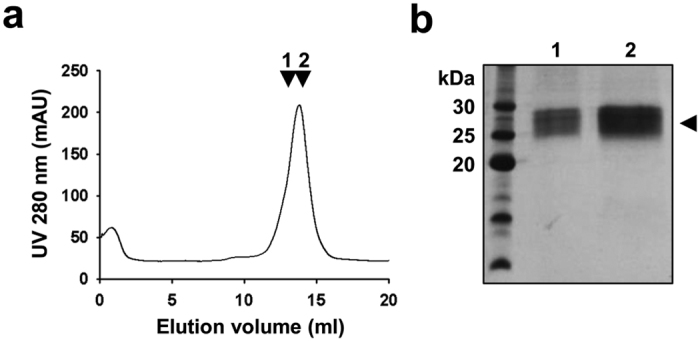
Purification of PfVIT by size-exclusion chromatography. (**a**) PfVIT was detected at 280 nm as a single major peak that eluted at 13.8 ml. The arrows correspond to peak fractions collected for analysis by SDS-PAGE. (**b**) Coomassie-stained SDS-PAGE analysis of peak PfVIT protein fractions from gel filtration chromatography. 10 μg of protein was loaded onto each lane.

Tag-cleaved PfVIT eluted as a single major peak at a volume of 13.8 ml from the size-exclusion column (Fig. 4a). Two other integral membrane proteins that we work with (and that are monomeric) – the *E. coli* GlpT (53 kDa)^21^ and multidrug efflux protein MdtM (45 kDa)^26^ - eluted at 11.6 ml and 12.0 ml, respectively on the same column. Therefore, an elution volume of 13.8 ml is consistent with a monomeric PfVIT-detergent-lipid complex. Analysis of the peak fractions by Coomassie-stained SDS-PAGE revealed the protein to be > 95% pure (Fig. 4b) and yields of pure PfVIT recovered from the size-exclusion column were typically in the ~0.5 mg range. MALDI TOF/TOF mass spectrometry analysis of purified, trypsin-digested protein confirmed that the band observed on SDS-PAGE was full length PfVIT.

### Purified PfVIT in detergent solution binds Fe^2+^ substrate with low micromolar affinity

PfVIT contains a single tryptophan and eleven tyrosine residues (Supplementary Fig. S2a). We hypothesised that binding of substrate to PfVIT might quench the intrinsic fluorescence of one or more of these residues and provide a method not only to judge the functional integrity of the protein after purification but also to assess the affinity of PfVIT for substrate. The apparent affinity of PfVIT for substrate was determined from the concentration of Fe^2+^ that produced half-maximum quenching of the protein fluorescence.

**Figure 5:**
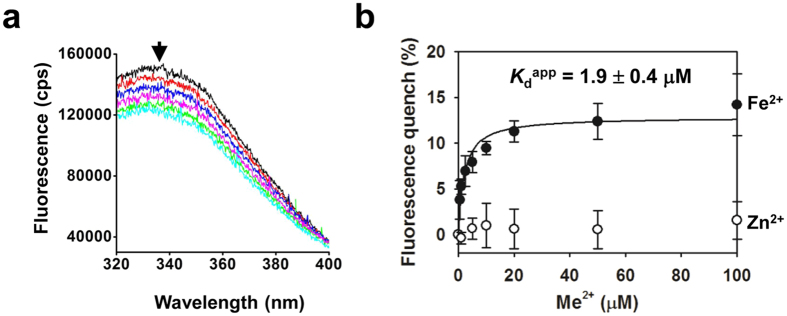
Substrate-binding affinity of purified PfVIT as measured by intrinsic protein fluorescence quenching. (**a**) Baseline-corrected fluorescence emission spectra of purified, detergent-solubilized PfVIT upon excitation at 280 nm. Fluorescence was measured at pH 6.0 in the absence or presence of the sulphate salt of Fe^2+^ at different concentrations (beginning at the top of the traces: 0, 0.5, 5, 20, 50 and 100 μM final concentration). (**b**) Substrate-binding affinity curve of Fe^2+^ binding (filled symbols) to purified PfVIT in FC-12 detergent solution. A control was provided by titration of Zn^2+^ (empty symbols) – a divalent metal cation that is not transported by PfVIT – into the protein solution. Data points and error bars represent the mean ± s.d. of three measurements.

The fluorescence emission spectrum (between 320 nm and 400 nm) of purified PfVIT in 0.01% w/v FC-12 detergent solution at pH 6.0 revealed maximal fluorescence emission to be centred at a wavelength of 337 nm when the protein was excited with light at 280 nm (Fig. 5a). Addition of Fe^2+^ to the protein solution induced a concentration-dependent quenching of the fluorescence emission without shifting the λ_max_. The apparent dissociation constant (*K*_d_^app^) for Fe^2+^ binding to the transporter was determined as 1.9 ± 0.4 μM (mean ± s.d) (Fig. 5b), indicating that purified PfVIT in FC-12 detergent solution retained substrate-binding capability and that our purification protocol resulted in functional protein.

As a control, we tested for binding of Zn^2+^ (a divalent metal cation that is not a substrate of PfVIT^17^) to the purified transporter. In contrast to the concentration-dependent fluorescence quenching observed when Fe^2+^ was titrated into the system and bound to the protein, addition of Zn^2+^ resulted in a small, nonspecific effect on the fluorescence emission at each concentration tested (Fig. 5b).

## Discussion

Vacuolar iron transport proteins play a vital role in compartmentalisation of labile Fe^2+^ in non-animal cells and thereby contribute to iron homeostasis in those systems. In yeast cells vacuolar uptake of Fe^2+^ is mediated by CCC1^13^, in plants by the CCC1 homologue vacuolar iron transporter 1 (VIT1)^10^,^19^ and in malaria-causing *P. falciparum* by the vacuolar iron transporter homologue PfVIT^17^. Despite the importance of VIT proteins to iron sequestration in many biological systems, little is understood of their mechanism of transport. Furthermore, although *Plasmodium* membrane proteins represent attractive potential drug targets, detailed investigation of their biochemical and biophysical properties has been hindered by lack of a source of isolated and functional recombinant proteins from the parasite^27^,^28^, exceptions being chloroquine resistance transporter PfCRT^29^, multi-drug resistance transporter PfMDR1^30^ and copper transport protein Ctr1^31^.

Due to financial and temporal factors, ease of use and genetic manipulation, *E. coli* is still a favoured host for heterologous expression of recombinant proteins. However, eukaryotic membrane proteins are often refractory to heterologous overexpression in *E. coli* and, in the case of *P. falciparum* proteins, rare codon usage and genome AT-richness can affect recombinant expression^32^. Use of a codon-optimised, synthetic gene that encoded PfVIT ligated into a vector containing the arabinose PBAD promoter, combined with systematic identification of appropriate culture conditions, enabled heterologous overproduction of functional PfVIT in *E. coli*. This approach may have wider application and enable functional overexpression of transporters from other plasmodial subcellular membranes.

Functional overexpression of PfVIT in the *E. coli* inner membrane permitted investigation of the substrate specificity profile of the transporter using a combination of assays of cell survival in the presence of externally added divalent metal cations and vesicular transport assays. *E. coli* cells that expressed PfVIT were rendered more tolerant towards the cytotoxic effects of Fe^2+^ but not the other first row transition metal ions Mn^2+^, Zn^2+^, Co^2+^ or Ni^2+^. This implies that PfVIT can distinguish between Fe^2+^ and other divalent metal cations. The origin of this metal selectivity is currently not understood, and whether PfVIT possesses one or more Fe^2+^-binding sites, and the identity of the coordinating ligands is unknown. However, as the ionic radii of all the first row transition metals tested are in a similar range, the observation that not all of them are transported by the protein suggests selectivity is not achieved solely on the basis of size. We therefore speculate that coordination number and geometry of the metal ion substrate are likely discriminating factors. A detailed description of substrate selectivity and binding by PfVIT will have to await the availability of high-resolution structural information on the protein bound to its metal cation substrate.

A previous study performed on PfVIT in a yeast system provided strong evidence for a ferrous iron transport function for the transporter^17^; as we have shown, the ability of heterologous expression of recombinant PfVIT in *E. coli* to confer protection against the cytotoxic effects of excess Fe^2+^ is consistent with such a function. The results of our vesicular transport assays and ICP-MS analysis provide additional persuasive evidence that, in the *E. coli* membrane environment, PfVIT functions as a Fe^2+^/H^+^transporter that utilises the electrochemical transmembrane gradient to catalyse a proton-driven antiport. Furthermore, the binding data obtained from purified PfVIT in detergent solution are consistent with the notion of PfVIT as a relatively low affinity (but high capacity) Fe^2+^ transporter^17^.

Protein phosphorylation plays an important regulatory role in the life cycle of the malaria parasite^33^ and, interestingly, PfVIT is postulated to possess four phospho-acceptor sites located on the N-terminal tail and the putative hydrophilic loop that connects TM2 and TM3 at amino acid residues S21, S122, S140 and T150^33^,^34^. This suggests phosphorylation/dephosphorylation of these residues as a possible regulatory mechanism for PfVIT-mediated transport. Indeed, other transporter proteins such as the biogenic monoamine transporters in mammalian brain utilise phosphorylation/dephosphorylation of the protein to regulate transport direction and activity^35^. Notably, we have demonstrated that PfVIT is functional in the *E. coli* membrane; this implies that *Plasmodium*-specific kinases/phosphatases are not required for the PfVIT transport function in the bacterial system although phosphorylation of the transporter might still be important in the malaria parasite for regulating function. Further interrogation of the functional and/or structural role of phosphorylation/dephosphorylation of PfVIT is now enabled by availability of purified recombinant protein.

The work presented here opens the door for a better understanding of the function and mechanism of a VIT family representative and, ultimately, it is hoped it will aid a fuller understanding of iron homeostasis in an important human pathogen.

## Methods

All growth media, antibiotics and reagents were purchased from Sigma-Aldrich (UK) unless stated otherwise.

### Synthesis and cloning of the PfVIT coding sequence

The 822 bp sequence (PlasmoDB gene ID: PF3D7_1223700) encoding the 273 amino acid residue PfVIT transporter from *Plasmodium falciparum* 3D7 was codon-optimised for expression in *Escherichia coli* (Supplementary Fig. S2) and synthesised using a commercially available service (GenScript, USA). Nco1 and EcoR1 restriction sites were introduced into the 5′ and 3′-ends of the synthetic DNA, respectively, and the optimised coding sequence was ligated into the multiple cloning site of a modified pBAD/*Myc*-His A expression vector (Invitrogen, UK) in frame with the plasmid-borne C-terminal His_6_ affinity tag to give a construct of 307 amino acid residues and molecular mass of 34.9 kDa. A thrombin-specific proteolysis site (LVPRGS) permitted subsequent cleavage of the *Myc*-His tag. The fidelity of the construct was verified by DNA sequence analysis.

### Protein overproduction

The plasmid construct was transformed into chemically competent *E. coli* LMG194 cells^36^ and an overnight culture grown from a single colony of freshly transformed cells was diluted 100-fold into twelve 5 l flasks, each containing 1 l of Luria Bertani (LB) broth supplemented with 100 μg ml^−1^ carbenicillin (Carbenicillin Direct, UK). These cultures were grown at 32 °C with 220 rpm shaking till the OD_600_ was 0.4, then the temperature was downshifted to 25 °C. At OD_600_ of 0.7 expression of PfVIT was induced by addition of 0.001% w/v L-(+) arabinose (Melford Laboratories Ltd., UK). Cells were grown for a further 4 h prior to harvesting.

### SDS-PAGE and western blot analysis

Expression levels of recombinant protein were analysed by western blot of DDM-detergent solubilised *E. coli* cell membranes. Solubilised protein samples of 80 μg (as determined by BCA assay) were loaded onto a bis-Tris gel of 15% acrylamide and resolved via SDS-PAGE before transfer to nitrocellulose membrane. The hexahistidine-tagged proteins were detected using HisProbe-HRP and SuperSignal West Pico Chemiluminescent Substrate (Thermo Scientific, UK) according to the manufacturer’s instructions.

### Metal sensitivity assays

Cultures of *E. coli* LMG194 transformed with either empty pBAD vector or pBAD vector that encoded PfVIT were grown from single bacterial colonies in LB medium supplemented with 100 μg ml^−1^ carbenicillin at 32 °C with shaking for 16 h then diluted 100-fold into fresh, antibiotic-containing LB broth. At OD_600_ of 0.4 the temperature was decreased to 25 °C and the cultures incubated until OD_600_ of 0.7. Expression of PfVIT was induced by addition of 0.001% w/v L-(+) arabinose and the cells grown for a further 2 h prior to use in qualitative assays on solid LB medium. For these assays, 2 μl aliquots from a 10^−3^ to 10^−6^ logarithmic dilution series of cultures that had OD_600_ adjusted to 1.0 were spotted onto LB agar plates containing 100 μg ml^−1^ carbenicillin and 0.001% w/v L-(+) arabinose to which 2 mM ascorbic acid and Fe^2+^ in the form of ammonium iron (II) sulphate at the stated concentrations were added. Plates were incubated for 24 h at 30 °C prior to imaging.

Quantitative assays of metal sensitivity were performed by counting the number of viable *E. coli* colonies after growth in liquid LB medium containing Fe^2+^, Mn^2+^, Zn^2+^, Co^2+^or Ni^2+^ at concentrations from 0 to 10 mM. The metal ions were provided in the form of their chloride salts, except for Fe^2+^ which was supplied to the medium as ammonium iron (II) sulphate in the presence of 2 mM ascorbic acid. Overnight cultures of *E. coli* LMG194 transformed with either empty pBAD vector or vector that encoded PfVIT were diluted 100-fold into LB broth containing 100 μg ml^−1^ carbenicillin and incubated at 32 °C with shaking until OD_600_ achieved 0.4. The temperature was then decreased to 25 °C and the cultures grown to OD_600_ of 0.7. Recombinant protein expression was induced for 2 h by addition of 0.001% (w/v) L-(+) arabinose. Subsequently, 100 μl of culture was diluted into 10 ml of LB broth supplemented with antibiotic, 0.001% (w/v) L-(+) arabinose, 2 mM ascorbate and the appropriate metal ion concentration in 14 ml polypropylene BD Falcon tubes (Corning, Mexico). The tubes were incubated for 16 h at 25 °C with gentle shaking. 100 μl aliquots were taken to perform serial dilutions in sterile Maximum Recovery Diluent (MRD). Diluted samples were then plated onto LB agar and the plates incubated overnight at 30 °C prior to performing colony counts.

### Quantitation of cellular iron content by ICP-MS

Quantitation of cellular iron content was performed using a method based on that described by Frawley *et al*.^37^. *E. coli* LMG194 cells transformed with either empty pBAD vector or pBAD vector that encoded PfVIT were grown in liquid LB medium as described above. After 2 h of induction, 5 ml aliquots of cells were pipetted into BD Falcon tubes containing LB broth, 2 mM ascorbate and ammonium iron (II) sulphate at the stated concentrations, and incubated for a further 30 min at 25 °C with gentle shaking. Cells were pelleted by centrifugation, washed twice with 10 ml MRD containing 1 mM EDTA and once with 1 ml of the same buffer, followed by centrifugation to remove the supernatant. The pelleted cells were air dried for 24 h then weighed. To prepare the samples for ICP-MS, cells were suspended in 1 ml of trace metal grade concentrated nitric acid (ROMIL Ltd., UK) and incubated at 80 °C for 45 min. Samples were centrifuged at high speed for 15 min, and 0.5 ml from each sample was transferred into 15 ml tube. The nitric acid solution was then diluted 1:10 by adding 4.5 ml MilliQ purified water. ICP-MS analysis was performed on an Agilent 7900 ICP-MS instrument by the Spectroscopy Group, Analytical Services, Almac, UK.

### Protein purification

PfVIT was purified using a protocol based on one described previously^38^. Harvested cells were resuspended in ice cold TBS (50 mM Tris-HCl, 100 mM NaCl, pH 7.5) and collected by centrifugation. All subsequent steps were performed at 4 °C. Cell pellet was resuspended in TBS buffer containing protease inhibitor cocktail (cOmplete™, Roche, UK) and 0.05% w/v β-mercaptoethanol to a final concentration of 0.2 g cells per ml. Resuspended cells were incubated under gentle agitation with 0.5 mg ml^−1^ lysozyme in presence of 1 mM EDTA for 30 min prior to treatment with 0.03 mg ml^−1^ DNase and 5 mM MgCl_2_. Complete cell breakage was achieved by three passages of the sample through a pre-chilled French pressure cell at 16,000 psi. Unbroken cells were removed by centrifugation at 20,000× g for 30 min, and the membrane pellet collected by ultracentrifugation. Isolated membranes were resuspended in solubilisation buffer (50 mM Tris-HCl pH 7.5, 300 mM NaCl, 10 mM imidazole) to a final concentration of 100 mg ml^−1^ and solubilised by incubation with 1.0% w/v dodecyl-β-D-maltopyranoside (DDM; Melford Laboratories Ltd., UK) for 1 h under gentle stirring. Solubilised membrane proteins were loaded onto an Econo-column (Bio-Rad Laboratories, UK) containing 0.5 ml of HisPur cobalt affinity resin (ThermoFisher Scientific, UK) and unbound protein was allowed to flow through under gravity. The column was then washed with 20 column volumes (CVs) of wash buffer (50 mM Tris-HCl pH 8.0, 300 mM NaCl, 10% v/v glycerol, 0.1% w/v DDM) containing 10 mM imidazole to remove non-specifically bound contaminants then incubated with ATP dissociation buffer (wash buffer containing 10 mM MgCl_2_, 5 mM ATP, 150 mM KCl) for 1 h to remove contaminating chaperonins. The resin was then washed with 20 CVs wash buffer supplemented with 30 mM imidazole prior to detergent exchange with 10 CVs of wash buffer containing 1% w/v Fos-Choline-12 (FC-12; Anatrace, UK). Subsequent to an additional wash step with buffer containing 0.1% w/v FC-12 the PfVIT target protein was cleaved from the column by incubation with 6 NIH units of thrombin (Novagen, UK). The volume of eluted protein was reduced to 1 ml using a 50 kDa MWCO centrifugal concentrator (Millipore, UK) then loaded onto a Superdex 200 10/300 GL gel filtration column (GE Healthcare, UK) equilibrated with 50 mM Tris-HCl pH 8.0, 300 mM NaCl, 10 mM imidazole, 5% v/v glycerol, 1 mM DTT, 0.1% w/v FC-12 and connected to an ÅKTA FPLC system. The chromatography run was performed at a flow-rate of 0.4 ml min^−1^ and protein was monitored by measuring absorbance at 280 nm.

### Protein quantitation

Protein was quantified by BCA assay (Thermo Scientific Pierce, USA) in accordance with the manufacturer’s instructions.

### Mass spectrometric analysis

Coomassie-stained SDS-PAGE gel bands were excised and cut into 1 mm cubes. These were then subjected to in-gel digestion, using a ProGest Investigator in-gel digestion robot (Digilab) using standard protocols^39^. Briefly, the gel cubes were destained by washing with acetonitrile and subjected to reduction and alkylation before digestion with trypsin at 37 °C. The peptides were extracted with 10% formic acid. For MALDI TOF/TOF analysis the digest solution (0.5 μL) was applied to the MALDI target along with alpha-cyano-4-hydroxycinnamic acid matrix (0.5 μL, 10 mg/mL in 50:50 acetonitrile:0.1% TFA) and allowed to dry. MALDI MS was acquired using a 4800 MALDI TOF/TOF Analyser (ABSciex) equipped with a Nd:YAG 355 nm laser and calibrated using a mixture of peptides. The most intense peptides (up to 15) were selected for MSMS analysis and the MS data analysed, using GPS Explorer (ABSciex) to interface with the Mascot 2.4 search engine (Matrix Science) and the MSMS data using Mascot 2.4 directly. Swiss-Prot (Dec 2012) or NCBInr (Aug 2013) databases were interrogated. No species restriction was applied. The data were searched with tolerances of 100 ppm for the precursor ions and 0.5 Da for the fragment ions, trypsin as the cleavage enzyme, assuming up to one missed cleavage, carbamidomethyl modification of cysteines as a fixed modification and methionine oxidation selected as a variable modification.

### Vesicular transport assays

All transport assays were performed at pH 7.2 on inverted vesicles generated from *E. coli* LMG194 cells that overexpressed recombinant PfVIT or, as a control, GlpT. Assays designed to detect changes in pH due to PfVIT-mediated Fe^2+^/H^+^ exchange utilised the fluorescent indicator acridine orange and were performed following a protocol described previously^40^.

For measurements of ferrous iron transport, Fe^2+^ substrate was provided in the form of its sulphate salt to final concentrations of 5 μM and 50 μM. To prevent oxidation and precipitation of the ferrous iron substrate in these experiments, all assay components were bubbled with nitrogen and the cuvette compartment of the fluorometer was maintained under constant nitrogen flush for the duration of the assay. In all transport assays the vesicles were energised by addition of 2 mM sodium lactate. In assays that tested for PfVIT-mediated transport of Fe^2+^ the proton electrochemical gradient was collapsed by addition of Triton X-100 to 0.07% (v/v) to disrupt the vesicles. In assays that tested for PfVIT-mediated transport of other divalent metal cations the proton electrochemical gradient was collapsed by addition of 100 μM CCCP.

### Substrate binding activity

Substrate-binding affinity of purified recombinant PfVIT for divalent metal ions in detergent solution was determined by intrinsic protein fluorescence quenching studies. Fluorescence experiments were performed at an excitation wavelength of 280 nm in a Fluoromax-4 fluorometer (Horiba UK Ltd) fitted with a thermostatically controlled cuvette holder. Purified PfVIT at a final concentration of 0.3 μM in FC-12 detergent solution (50 mM Bis-Tris pH 6.0, 100 mM NaCl, 5% v/v glycerol, 0.01% w/v FC-12) was titrated with the sulphate salt of Fe^2+^ until maximal fluorescence quenching at the emission maximum of 337 nm was achieved. All buffers were bubbled with nitrogen to remove dissolved oxygen prior to measurements of fluorescence and the cuvette compartment of the fluorometer was maintained under constant nitrogen flush for the duration of each experiment. As a control, the protein solution was titrated with the chloride salt of Zn^2+^. All measurements were performed in triplicate at 25 °C. The data underwent nonlinear regression analysis to calculate the apparent dissociation constant, *K*_d_^app^, for binding.

## Additional Information

**How to cite this article**: Labarbuta, P. *et al*. Recombinant vacuolar iron transporter family homologue PfVIT from human malaria-causing *Plasmodium falciparum* is a Fe^2+^/H^+^ exchanger. *Sci. Rep.* **7**, 42850; doi: 10.1038/srep42850 (2017).

**Publisher's note:** Springer Nature remains neutral with regard to jurisdictional claims in published maps and institutional affiliations.

## Supplementary Material

Supplementary Information
