# Supplementary material for: Recombinant vacuolar iron transporter family homologue PfVIT from human malaria-causing Plasmodium falciparum is a Fe2+/H+exchanger
Source: Sci Rep. 2017 Feb 15;7:42850. doi: 10.1038/srep42850 (PMC5309874; doi:10.1038/srep42850)
Supplement: Supplementary Information [file srep42850-s1.pdf]

Supplementary Information

for:

Recombinant vacuolar iron transporter family homologue PfVIT from human malaria-causing *Plasmodium falciparum* is a Fe^{2+}/H^+ exchanger

Paola Labarbuta[1], Katie Duckett[1], Catherine H. Botting[2], Osama Chahrour[3], John Malone[3], John P. Dalton[1] and Christopher J. Law[1,*]

[1]School of Biological Sciences, Medical Biology Centre, Queen's University Belfast, Belfast BT9 7BL, United Kingdom.

[2]School of Biology, Biomolecular Sciences Building, University of St Andrews, North Haugh, St Andrews, Fife KY16 9ST, United Kingdom.

[3]Spectroscopy Group, Analytical Services, Almac, 20 Seagoe Industrial Estate, Craigavon BT63 5QD, United Kingdom.

*Corresponding author: email: c.law@qub.ac.uk; Tel: 028 90972071; Fax: 028 90975877

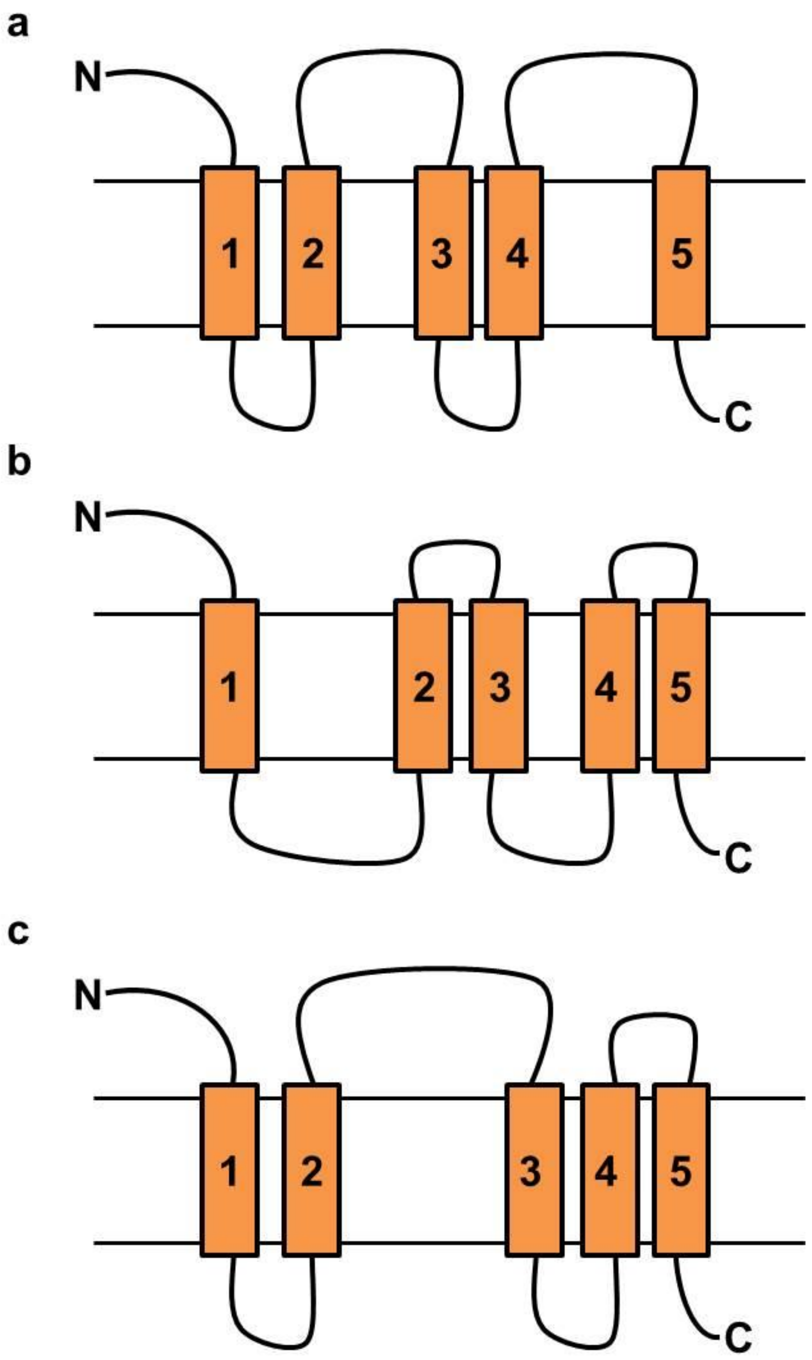

Figure S1 – Putative arrangement of transmembrane helices (represented as orange rectangles) in different VIT family representatives. **(a)** 2+2+1 arrangement. **(b)** 1+2+2 arrangement. **(c)** 2+3 arrangement. In all VIT proteins the extended N-terminal is predicted to be located in the cytoplasm and the C-terminal in the compartmental space.

a

```
                10         20         30         40         50         60         70
       ....|....| ....|....| ....|....| ....|....| ....|....| ....|....| ....|....|
PfVIT  MVSKKTIEAR KAYYNEDVVL SKEAHDFYHN LDKHGENHNL DKDNLKTIIF GSLDGIITIF AIVSGCVGAK

                80         90        100        110        120        130        140
       ....|....| ....|....| ....|....| ....|....| ....|....| ....|....| ....|....|
PfVIT  ITPTQVIIIG IGNLFANAIS MGFSEYTSST AQRDFMLAEK KREEWEIENC PSEEKQEMID IYMNKYKFDS

               150        160        170        180        190        200        210
       ....|....| ....|....| ....|....| ....|....| ....|....| ....|....| ....|....|
PfVIT  EDARNLVEIT FRNKNFFLEH MMSEELGLIV TNEDKNECLK KGIIMFLSFA VFGIIPLSAY VAYTVFFGYT

               220        230        240        250        260        270
       ....|....| ....|....| ....|....| ....|....| ....|....| ....|....| ...
PfVIT  DYTTSFLVVF ISTLTTLFIL GLFKSQFTNQ KPITCALYMV LNGMIAGMVP FLLGVVLKNN ISE
```

b

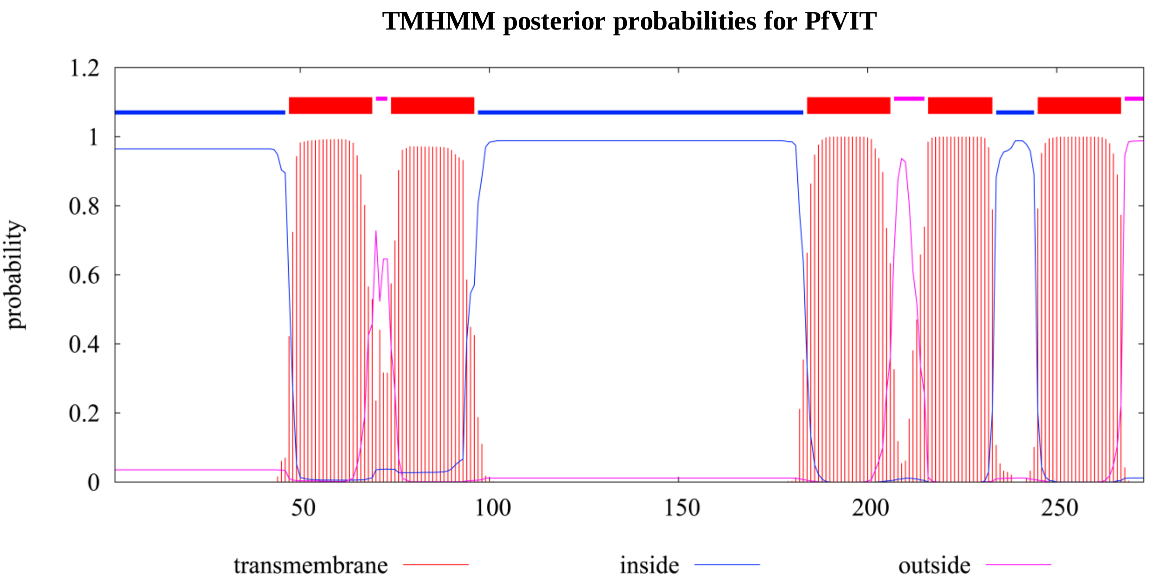

Figure S2 – **(a)** Primary sequence of PfVIT (UniProtKB - Q81510). **(b)** Predicted topology of PfVIT using TMHMM Server v.2.0 [1]. PfVIT is predicted to contain 5 transmembrane-spanning helices organised in a '2 + 3' arrangement connected by a long and hydrophilic, cytoplasmic loop region. The N- and C-terminals of PfVIT are predicted to reside in the cytoplasm and vacuole (or ER) interior, respectively.

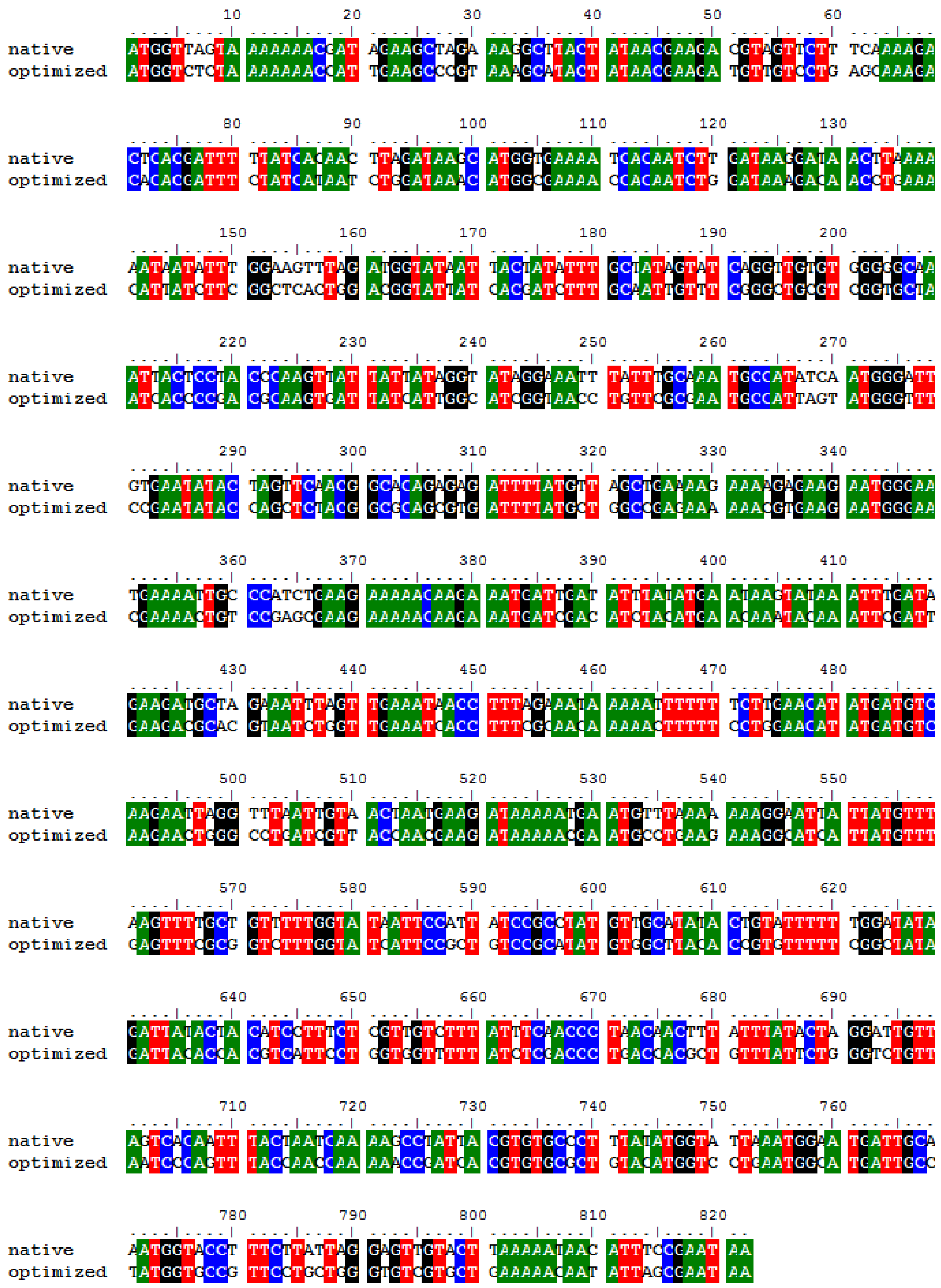

Figure S3 – Native and synthetic *pfvit* nucleotide sequences. Synthetic *pfvit* was codon-optimised for heterologous expression of the gene product in *E. coli*.

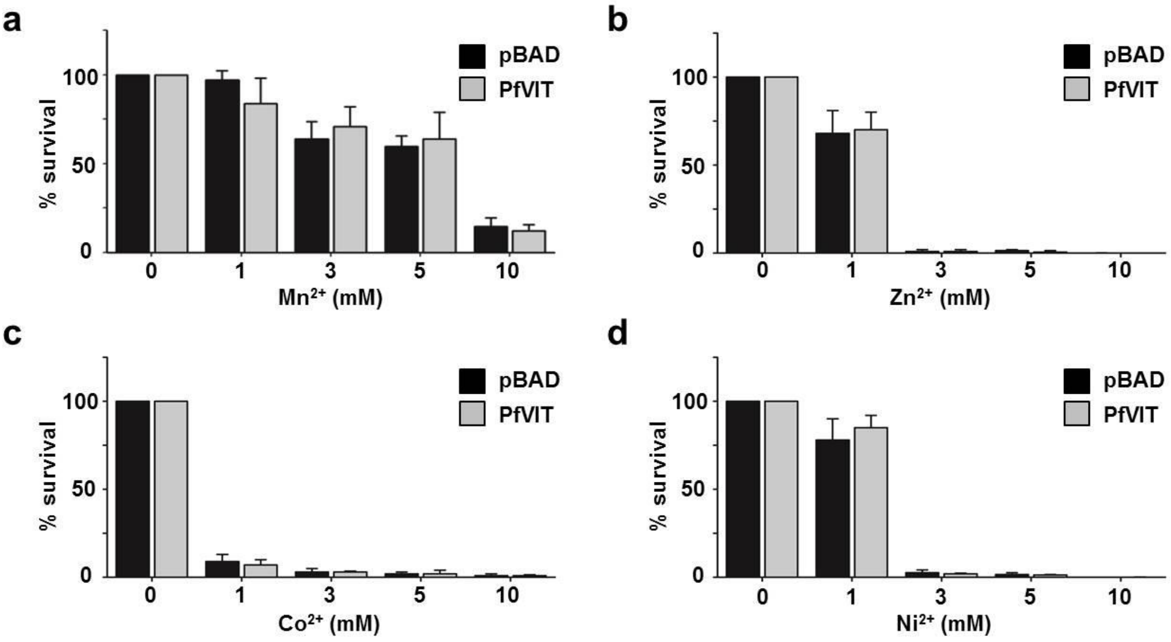

Figure S4 – The effect of functional expression of PfVIT on the viability of *E. coli* LMG194 cells in liquid LB medium that contained **(a)** Mn^{2+} **(b)** Zn^{2+} **(c)** Co^{2+} or **(d)** Ni^{2+} at the concentrations indicated. After 16 h incubation with shaking at 25°C cell viability was determined by colony-forming unit (cfu) counts. The cfu counts were expressed as a percentage of those of control cultures that were grown in liquid medium that contained no additional Me^{2+}. Bars and error bars represent the mean ± s.d. of three separate measurements.

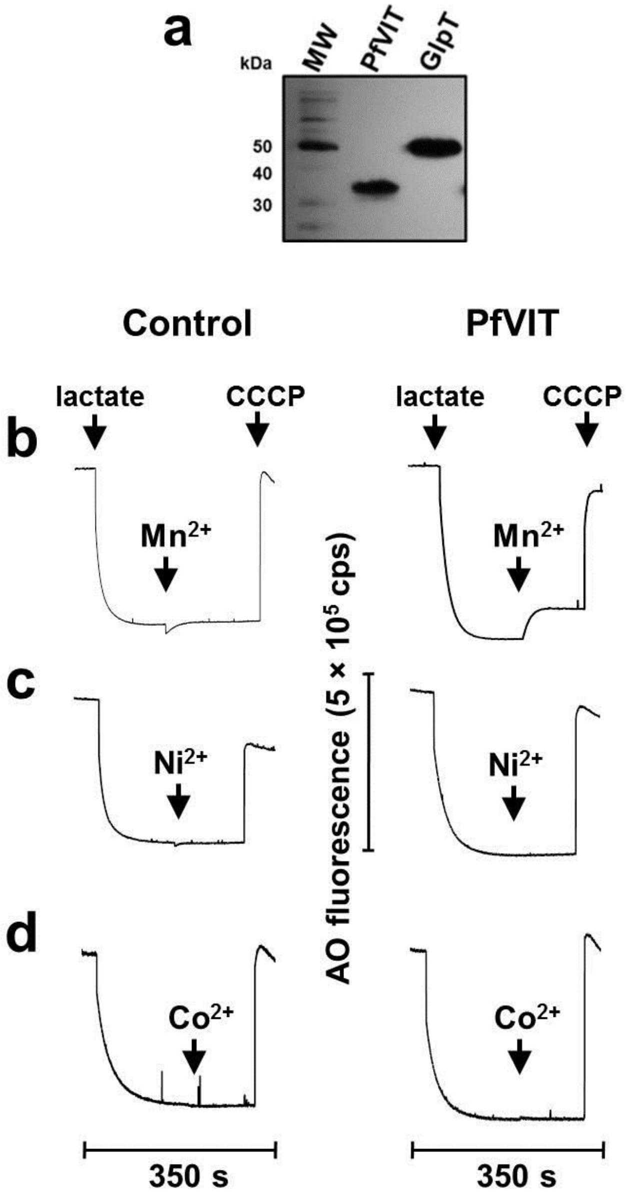

Figure S5 – Vesicular transport assays to test substrate specificity of PfVIT. **(a)** Western blot analysis of expression levels of PfVIT and GlpT control in inverted vesicles. 10 μg of total membrane protein from DDM-detergent solubilised vesicles was loaded onto each lane of the gel and protein was detected using HisProbe-HRP. PfVIT mediated transport of **(b)** Mn^{2+} when the metal cation was added at a non-physiologically relevant concentration of 5 mM to inverted vesicles. Addition of **(c)** Ni^{2+} or **(d)** Co^{2+} to the same final concentration of 5 mM did not result in any

detectable transport. Measurements were performed by monitoring the fluorescence dequench of acridine orange upon addition of divalent metal ions to inverted vesicles prepared from *E. coli* cells that overproduced recombinant PfVIT (right traces) or, as a control, GlpT (left traces). Respiration-dependent generation of ΔpH (acid inside) was established by addition of lactate as indicated. Addition of CCCP at the time indicated completely dissipated the proton gradient. Fluorescence intensity was measured in counts per second (cps).

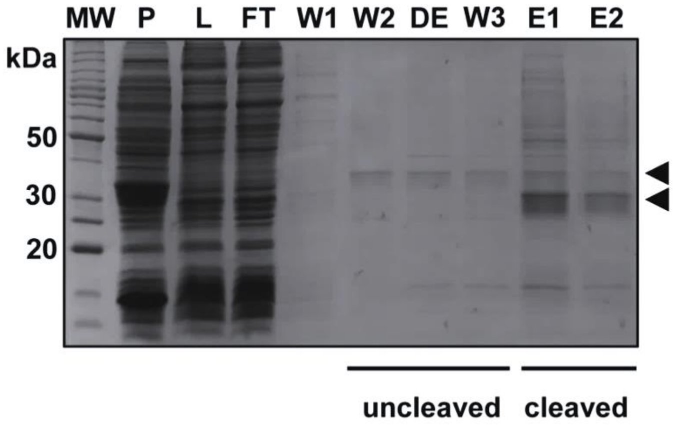

Figure S6 – Coomassie-stained SDS-PAGE of Co^{2+}-affinity resin purification of PfVIT that included a detergent exchange step (to replace DDM detergent with FC-12) and an incubation step with ATP to remove chaperonin contamination. Samples were incubated in SDS-PAGE sample loading buffer for 10 min at room temperature prior to loading onto the gel. Gel lanes were loaded with molecular weight marker protein marker (M), membrane pellet fraction (P), DDM-soluble fraction (L), column flow through (FT), wash step (W1), ATP dissociation step to remove chaperonin (W2), detergent exchange (DE), final wash (W3), and thrombin-cleaved fractions (E1

and E2). The lower arrow indicates PfVIT which has had the hexahistidine affinity tag removed by thrombin and the upper arrow indicates uncleaved PfVIT protein.

References

[1] Sonnhammer, E.L.L., von Heijne, G. & Krogh, A. A hidden Markov model for predicting transmembrane helices in protein sequences*.* *In* Proc. of Sixth Int. Conf. on Intelligent Systems for Molecular Biology, p 175-182 . Eds. Glasgow. J *et al*. AAAI Press, Menlo Park, CA (1998)
